# The Poor Man's Home.—XII

**Published:** 1897-11-06

**Authors:** 


### Modern Sociology.

### THE POOR MAN'S HOME.—XII.

### Master and Man (*continued*).

One of the best known experiments in the housing of workmen by their employer is to be found in France. Its fame rests, however, more on the fact that it was erected as a socialistic experiment than on its merits as a dwelling. In fact, from the point of view of most of us, it fails there, in that it rather discourages that sense of family privacy which it is the aim of most builders for the poor to preserve. But M. Godin, the founder of the Familistère at Guise, was not only a shrewd man of business—in fact, he seems to have been that in spite of himself—but a philosophical socialist and a speculative enthusiast. To aid in bringing about that "solidarity of the human race" which is the ideal of such thinkers, he built this great barrack (for such it seems to the non-socialistic eye) for his workmen to live in. The left wing of the first group of block dwellings was begun in 1859, and was finished in 1861. The central part was put up immediately after, but the right wing was not erected till 1877. A second block was put up in 1882, and a third between 1883 and 1885. The industrial town, for such it is, is completed by buildings containing stores, a nursery, a theatre, two schools, a public laundry, and baths. In the first block there are 299 tenements, 12 of which consist of 1 room, 201 of 2, 75 of 3; while there is also a single tenement of 4 rooms, and 6 of 5, 3 of 7, and 1 of 8 rooms. The intention was to house all grades of workers under one roof, and bind them to each other by common interests and responsibilities. Whether or not the experiment proved as successful as M. Godin anticipated, we cannot say. Certainly he himself never swerved from his principles; but the fact remains that in the later buildings the smaller tenements are in a large majority. Indeed, there is only one of more than three rooms. The first block, is however, much larger than the others.

The method by which rents are computed renders it difficult for us to compare those of the Familistère with those of other places. They are estimated, not by accommodation, but by floor space. Position, however, affects the value of superficies. Thus, in the left wing and the centre of the first block rents vary from 2½d. to nearly 3½d. per square yard (it is impossible to give the exact value of the respective centimes in English money), while in the right wing they may fall to a little over 2d. per square yard. These are the lowest in the whole Familistère, those of the remaining blocks being, within a fraction, the same as the left wing of the first block. The total rent-roll amounts to about £4,440. It is considered a privilege to live in the Familistère, and various benefits in the way of pensions are secured to the residents, in proportion to the time they have been there. This, however, does not concern our present subject. It cannot be said that the Familistere, in spite of the goodwill that went to its foundation, is an altogether satisfactory dwelling. The sanitary arrangements, as is not uncommon in France, leave much to be desired. There are only 99 privies—a form of closet which would at best hardly satisfy a British mind—to a population of about 1,800. These are cleaned out once a year; but liquid sewage is run off immediately into a river which passes through the grounds. Water is supplied from artesian wells, from which it rises to nearly the height of the house, and is distributed to reservoirs, from which it is conveyed by pipes to the different floors. The reservoirs hold about 2,200 gallons.

In Germany there is a very fine example of what employers can do in the town of Essen, which, as it now is, may be regarded as the creation of Messrs. Krupp, the famous makers of big guns. Their ideal is the opposite of M. Godin's. They have built small self-contained houses for their workmen, although there are also some flats in block dwellings, as well as lodging-houses for unmarried workmen. Moreover, they are willing to advance money to such workmen as desire to build houses for themselves. The privacy and individuality of the family are as much encouraged here as they are discouraged in the French experiment. This is so much so that privacy is preferred to ventilation in the tenement blocks. Two dead walls, at right angles to each other, pass through the centre of each block, dividing it so that there are only four families on each floor. This makes the flats much like "back-to-back" houses, in which there is no through ventilation. Each family has a privy for its own use, but these are of a rather primitive nature, being, indeed, only a pit. The firm has, however, provided an abundant water-supply for the town. There are also small block houses intended for four families in each building. The wisest plan on which to accommodate this number of families would probably have been to have divided each of the two storeys to which it extends into two, and given half a flat to each family. Each would thus have been able to obtain through ventilation from front to back. This method has not been followed. Instead, we have again the cross walls, and each family has a two-storey corner of the building of the house; perfectly isolated, indeed, but by no means perfectly ventilated. In these houses the kitchens measure 6 ft. 9 in. by 9 ft. 6 in., the living-rooms 16 ft. 5 in. by 13 ft. 1 in. One bedroom is 16 ft. 5 in. by 10 ft. 1 in., and the other 9 ft. 10 in. by 9 ft. 2 in. The rents of these houses run from £8 to £9 per annum. A workman who wishes to own his house must be able to put down at once 300 marks, a sum equivalent to £14 7s. 6d., must give a mortgage on the property he means to erect, and provide for the repayment of the sum he desires to borrow by regular instalments, spread over a period of 25 years. These conditions being fulfilled, the necessary money is lent at 3 per cent. interest. Anyone claiming the advantage of these terms must have been in the employ of the firm for at least three years; he must be between the ages of 25 and 50; he must be married; and he must have completed his term of military service. The firm provides an architect; but the owner can build in any style he pleases, so long as the general building regulations are observed.

---

The cost of collecting money is often very large. This is shown in respect to rather a novel method of securing subscriptions towards a Cardiff Infirmary cot, which it is intended to endow in honour of the Queen's long reign. It appears that the committee sent out 200 collection-boxes to the hotels in the town, offering three prizes, amounting to £20, for the largest collection. These 200 boxes produced only £105 17s., the first prize falling to the collector of £15 3s.

